# Evaluation of Immunomodulatory Effects of *Fusarium* Mycotoxins Using Bacterial Endotoxin-Stimulated Bovine Epithelial Cells and Macrophages in Co-Culture

**DOI:** 10.3390/genes14112014

**Published:** 2023-10-27

**Authors:** Umesh K. Shandilya, Ankita Sharma, Ran Xu, Maria Malane M. Muniz, Niel A. Karrow

**Affiliations:** Department of Animal Biosciences, University of Guelph, 50 Stone Rd. E., Guelph, ON N1G2W1, Canada; ushand@uoguelph.ca (U.K.S.); ankitas@uoguelph.ca (A.S.); rxu02@uoguelph.ca (R.X.); mmagalha@uoguelph.ca (M.M.M.M.)

**Keywords:** co-culture, cytokines, inflammatory response, macrophages, mycotoxin

## Abstract

Mycotoxins are secondary metabolites produced by a variety of fungi that contaminate animal food and feeds and are capable of inducing a wide range of toxicities. Predictive in vitro models represent valuable substitutes for animal experiments to assess the toxicity of mycotoxins. The complexities of the interactions between epithelial and innate immune cells, vital for upholding barrier integrity and averting infections, remain inadequately understood. In the current study, a co-culture model of bovine epithelial cells (MAC-T) and macrophages (BoMac) was used to investigate the impact of exposure to *Fusarium* mycotoxins, namely deoxynivalenol (DON), zearalenone (ZEN), enniatin B (ENB), and beauvericin (BEA), on the inflammatory response elicited by the bacterial lipopolysaccharide (LPS) endotoxin. The MAC-T cells and BoMac were seeded on the apical side of a Transwell membrane and in the lower chamber, respectively, and mycotoxin exposure on the apical side of the membrane was carried out with the different mycotoxins (LC20; concentrations that elicited 20% cytotoxicity) for 48 h followed by an LPS immunity challenge for 24 h. The culture supernatants were collected from the basolateral compartment and these samples were submitted for cytokine/chemokine multiplex analysis. RNA-Seq analysis was performed using total RNA extracted from the MAC-T cells to acquire a more detailed insight into their cellular functions. The multiplex analysis indicated that IFN-γ, IL-1α, IL-8, and MCP-1 were significantly induced post-DON treatment when compared to control cells, and levels of IL-1α and IL-8 were enhanced significantly in all mycotoxin-treated groups post-LPS challenge. Analysis of the sequencing results showed that there were 341, 357, and 318 differentially expressed MAC-T cell genes that were up-regulated in the DON, ENB, and BEA groups, respectively. Gene ontology and pathway analysis revealed that these DEGs were significantly enriched in various biological processes and pathways related to inflammation, apoptosis signaling, and Wnt signaling. These results provide a comprehensive analysis of the co-culture cytokine/chemokine production and MAC-T cells’ gene expression profiles elicited by *Fusarium* mycotoxins, which further contributes to the understanding of early endotoxemia post-mycotoxin exposure.

## 1. Introduction

Mycotoxins are structurally diverse toxic secondary metabolites produced by fungi which frequently occur in feed and food worldwide. Contamination of livestock feeds with mycotoxins poses a worldwide problem, with an acknowledged negative effect on both human and animal health, and significantly impacts agricultural economics and international trade. Extensive evidence has shown that mycotoxins exert broad immunomodulatory effects on both cellular- and humor-based immune responses, resulting in a reduced host resistance to infectious diseases. For example, in mice, repeated exposure to the trichothecene T-2 toxin derived from *Fusarium* spp. has been observed to diminish their resistance to a range of infections, including *Mycobacterium bovis*, *Salmonella typhimurium*, *Listeria monocytogenes*, *Staphylococcus aureus*, and herpes simplex virus type 1 [1,2]. In a similar vein, the ingestion of this mycotoxin has been found to lower the resistance of rabbits to Aspergillus fumigatus infections [3]. Several reports show that mycotoxins are able to affect the innate immune system at different levels. For example, the *Fusarium* mycotoxins deoxynivalenol (DON) and fumonisin B1 (FB1) have been reported to directly affect the viability of phagocytic cells such as macrophages. Moreover, these mycotoxins can induce cellular apoptosis, inhibit proliferation, and impair the function of epithelial cells in vitro and in vivo [4,5], and can affect the expression of cytokines and chemokines, leading to immunosuppression and increased susceptibility to infections [6]. Several mycotoxins are also able to modulate the production of cytokines in different organs and/or cell types [7,8].

Various in vitro cell culture models have been established to mimic tissues or microenvironments to study various mechanistic aspects of the host inflammatory response [9,10,11]. In contrast to intricate in vivo models, diverse forms of cell culture offer a simplified, cost-effective, and targeted approach. They also require fewer, or even no, animals and facilitate the high-throughput analysis of biomaterials. In vitro co-culture systems have also been used to study the interactions between cell populations because they provide more natural interactions between populations, including at cellular and subcellular levels; for example, cell–cell interactions, cell growth, and to elucidate metabolism and molecular pathways. Moreover, this approach helps to reduce and replace animal experiments [12].

Lipopolysaccharide (LPS) endotoxin, which is a potent pathogen-associated molecular pattern (PAMP) making up the cell membrane of Gram-negative bacteria, is commonly used to activate cells grown in vitro to simulate an inflammatory response associated with bacterial infections. LPS is primarily recognized by the host cell membrane pattern recognition receptor (PRR) toll-like receptor 4 (TLR4), and contributes to several livestock pathologies, including mastitis [13], acidosis [14], and gut leakage due to heat stress [15], and human pathologies, such as systematic inflammatory response syndrome and sepsis [16].

Despite the establishment and recognition of the importance of co-culture systems, very few data are available concerning the effects of toxins on cellular functions cultured under these conditions, and we are unaware of any mycotoxin exposures that are carried out using co-culture systems. In this study, bovine mammary epithelial cells (MAC-T cell) and macrophage cell lines (BoMac) were grown in a co-culture system and exposed to various *Fusarium* mycotoxins followed by a bacterial LPS immune challenge. We hypothesized that mycotoxin exposures would cause specific alterations in the cellular functions assessed by RNA Sequencing (RNA-Seq) and cytokine/chemokine analyses, which could contribute to an increased susceptibility to infection.

## 2. Materials and Methods

### 2.1. Mycotoxins

Mycotoxin standards, namely deoxynivalenol (DON), zearalenone (ZEN), enniatin B (ENB), and beauvericin (BEA), were purchased from Sigma-Aldrich (St. Louis, MO, USA). These mycotoxins were dissolved in dimethyl sulfoxide (DMSO) to final stock concentrations of 5 mg/mL and stored at −20 °C until further dilution for subsequent experiments. The mycotoxin concentrations needed for the co-culture were prepared by making dilutions of the stock concentrations in the cell culture medium. 

### 2.2. Maintenance of Bovine MAC-T and BoMac Cell Lines

The bovine epithelial cell line (MAC-T) was cultured in a T25 culture flask (Corning, Tewksbury, MA, USA) according to the previous protocol [2] in Dulbecco’s modified Eagle medium (DMEM), supplemented with 10% fetal bovine serum and penicillin/streptomycin (100 U/mL; Invitrogen, Waltham, MA, USA; Thermo Fisher Scientific, Inc., Waltham, MA, USA), and maintained in an incubator at 37 °C with 5% CO_2_.

The bovine macrophage (BoMac) cell line [17] was cultured in a T25 culture flask (Corning, Tewksbury, MA, USA) using Roswell Park Memorial Institute 1640 medium (RPMI 1640; Invitrogen, Burlington, ON, Canada). The culture medium was supplemented with 2.0 mM L-glutamine, 10% heat-inactivated fetal bovine serum (FBS; Invitrogen), 2.5 mM HEPES buffer (Invitrogen), 1 mM Sodium Pyruvate (Invitrogen), and 1% Antibiotic-Antimycotic (containing 100 units/mL of penicillin, 100 µg/mL of streptomycin, and 0.25 µg/mL of amphotericin B; Invitrogen). The cells were maintained at 37 °C in an atmosphere containing 5% CO_2_.

### 2.3. Co-Culture Model Construction

**Figure 1:**
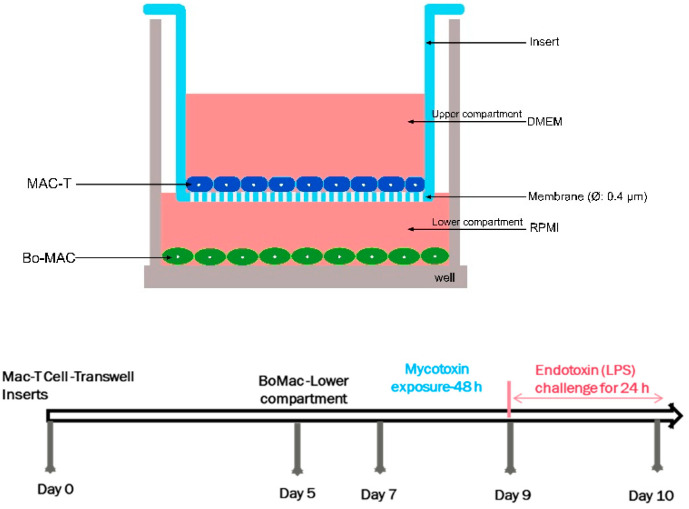
Schematic representation of the constructed in vitro co-culture system of bovine mammary epithelial cells (MAC-T) and macrophages (BoMac).

An in vitro non-contact co-culture model was constructed with bovine MAC-T and BoMac cell lines (Figure 1). Transwell inserts (0.4 μm pore size; Falcon, Corning, New-York, NY, USA) that were fitted to 24-well plates were seeded with MAC-T cells on the apical side, and BoMac occupied the lower chamber. The MAC-T cells were initially seeded at a density of 10^5^ cells/insert and were cultured for 5 days under normal culture conditions with the appropriate culture medium. Then, the cell culture inserts containing MAC-T cells were placed into the 24-well culture plates (Falcon) containing BoMac cells and seeded at a density of 2 × 10^5^ cells/mL. Both co-cultured MAC-T and BoMac cells were supplied with respective media and allowed to become established for 48 h before mycotoxins exposure studies.

### 2.4. Mycotoxin Exposure and LPS Challenge

Once the co-culture model was established, the upper compartments of the inserts were exposed to LC20 concentrations (a concentration that was cytotoxic to 20% of the cells) of different mycotoxins (DON-3.4 μM, ENB-29.9 μM, BEA-11.3 μM, ZEA-37.7) in media for 48 h. After mycotoxin exposures, *Escherichia coli* LPS (1 mg/mL, Sigma-Aldrich, Oakville, ON, Canada) was added to the upper compartment for 24 h. The final volume of the medium was 500 μL in the bottom of each well (named the lower compartment), and 200 μL in the hanging inserts (upper compartments). Both MAC-T and BoMac cells were maintained in their respective medium as mentioned above.

### 2.5. Multiplex Cytokine/Chemokine Analysis

The culture supernatants were collected from the lower compartments of the co-culture system and then sent to Eve Technologies (Calgary, AB, Canada) for multiplexing using the Luminex 100 system [18,19]. The cytokines examined in this study comprised IFN-γ, IL-1α, IL-6, vascular endothelial growth factor (VEGF-α), TNF-α, IL-10, and IL-36-α, while the chemokines included CXCL8 (IL-8), CCL3 (MIP-1α), CCL4 (MIP-1β), and CXCL10 (IP-10). 

### 2.6. RNA Isolation and Sequencing

Total RNA was extracted from the MAC-T cells of the co-culture system 48 h after mycotoxin exposure using the RNeasy Mini Kit (Qiagen, Valencia, CA, USA) in accordance with the manufacturer’s instructions. The concentration and purity of the RNA samples were determined based on A260/280 nm ratios using the BioTek Cytation-5 Spectrophotometer (Agilent, CA, USA). The integrity of the RNA samples was assessed using the Agilent 2100 Bioanalyzer (Agilent, Santa Clara, CA, USA), and samples with an RNA integrity number (RIN) greater than 7 were selected for RNA-Seq. The samples were outsourced to Genewiz (Burlington, MA, USA) to create cDNA libraries and then sequenced using the HiSeq 2500 sequencer (Illumina, San Diego, CA, USA).

### 2.7. RNA-Seq Analysis

Quality control for the data was conducted using the CLC Genomics Workbench software version 12.0 (CLC Bio, Aarhus, Denmark). This quality control assessment involved the examination of several parameters, including GC content, ambiguous base content, Phred score (Phred < 30), base coverage, nucleotide contributions, and over-represented sequence parameters [20]. The paired-end sequence reads were aligned to the bovine genome references (Bos_Taurus.ARS-UCD1.2.101) following the previously proposed guidelines [20]. To facilitate comparisons between groups, the transcripts were quantified in reads per kilobase per million mapped reads (RPKM) and transformed to a log2 scale [21]. The analyses of differential mRNA expressions were carried out using CLC Genomics Workbench 12.0 (CLC Bio, Aarhus, Denmark). The statistical analysis employed the “Exact Test” for comparing two groups and included two parameters related to dispersion estimation: a total count filter cut-off >5.0 and an estimation of tag-wise dispersions. Furthermore, differentially expressed genes (DEGs) between the two groups were defined based on specific criteria, including a *p*-value < 0.0001, FDR (False Discovery Rate) < 0.05, and a fold change (FC) exceeding |2| [22]. To assess the overlap of DEGs between different treatments, Venn diagram analysis was utilized (http://bioinformatics.psb.ugent.be/webtools/Venn/; accessed on 21 December 2021).

Discovery of DEGs in each pairing (DON vs. LPS-Control, ENB vs. LPS-Control, and BEA vs. LPS-Control) was accomplished using a Poisson distribution test [23]. An FDR ≤ 0.05 and FC (log2) ≥ 1 were used as thresholds to evaluate the significance of the DEGs.

### 2.8. Gene Ontology and Pathway Analyses

To systematically analyze the DEG functions of our data, the DEGs were uploaded onto PANTHER for gene ontology (GO) [24] and Kyoto Encyclopedia of Genes and Genomes (KEGG) pathway analyses [25,26]. The GO terms and pathways with a *p* < 0.05 were considered significantly enriched.

### 2.9. Statistical Analysis

To compare differences in the cytokines/chemokines concentrations of culture supernatants, the values were analyzed using a two-way ANOVA test followed by Bonferroni test (GraphPad Prism Software 9.3.1, Boston, MA, USA), and a *p*-value ≤ 0.05 was considered statistically significant. All data (*n* = 4 per treatment) were presented as the mean ± standard error of the mean (SEM). 

## 3. Results

### 3.1. Production of Cytokines/Chemokines

#### 3.1.1. Impact of Mycotoxin Treatment

**Figure 2:**
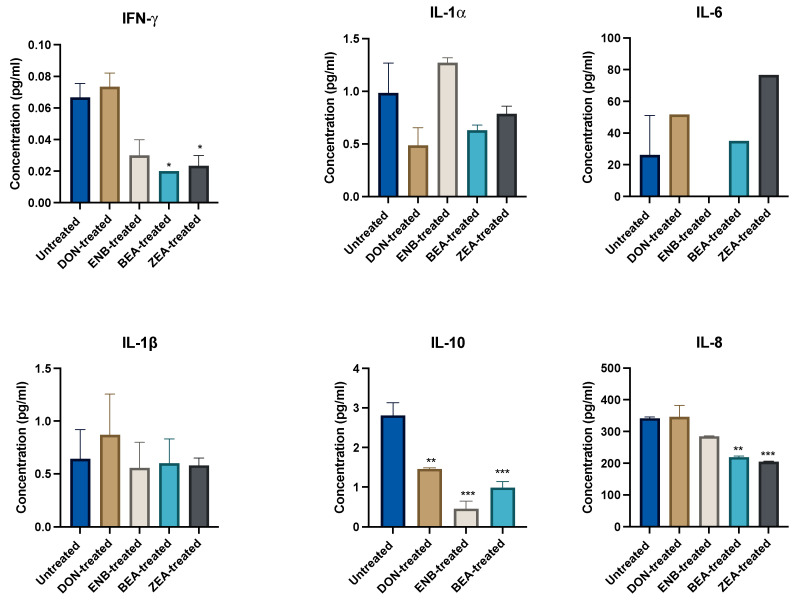
Effect of mycotoxin exposure for 48 h on cytokine and chemokine production in the culture supernatant of the co-culture system (Bovine MAC-T and macrophages) compared to the untreated control (no mycotoxin exposure) group. Data are expressed as mean (pg/mL) and SEM, and significant differences are denoted by * *p* < 0.05, ** *p* < 0.01 and *** *p* < 0.001. Includes results from deoxynivalenol (DON), enniatin B (ENB), beauvericin (BEA), and zearalenone (ZEN) exposure.

**Figure 3:**
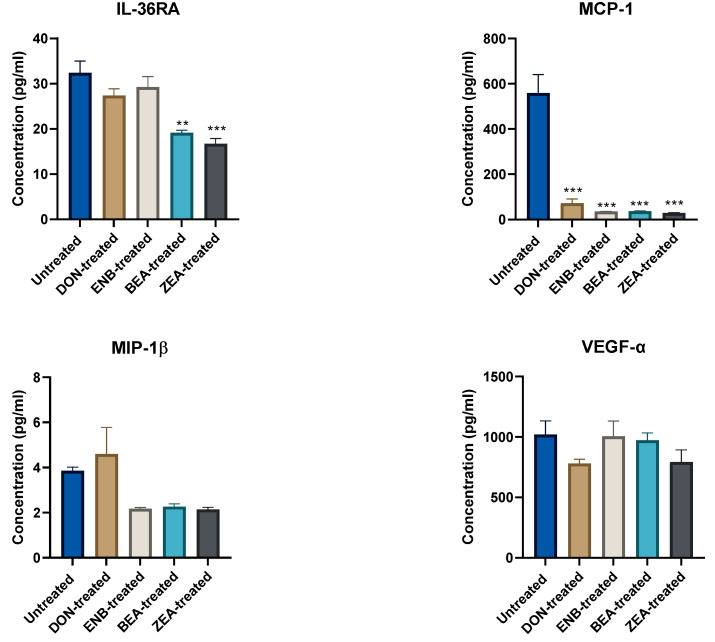
Effects of mycotoxin exposure for 48 h on cytokine and chemokine production in the culture supernatant of the co-culture system (Bovine MAC-T and macrophages) compared to the untreated control (no mycotoxin exposure) group. Data are expressed as mean (pg/mL) and SEM, and significant differences are denoted by ** *p* < 0.01, and *** *p* < 0.001. Includes results from deoxynivalenol (DON), enniatin B (ENB), beauvericin (BEA), and zearalenone (ZEN) exposure.

To assess the effect of mycotoxin treatment alone on cytokine production, comparisons were drawn between the mycotoxin-treated groups that had not been stimulated with LPS and the untreated LPS-negative control group, as depicted in Figure 2 and Figure 3. Notably, mycotoxins BEA and ZEA significantly diminished IFN-γ, IL-8, IL-10, and IL-36RA levels compared to the untreated group (*p* ≤ 0.05). In addition, DON also inhibited IL-10 significantly. Moreover, a consistent reduction in MCP-1 levels was observed across all mycotoxin treatment groups.

#### 3.1.2. Effect of LPS-Positive Control Versus Untreated LPS-Negative Control Group

In the control group exposed to LPS and devoid of mycotoxin exposure, LPS stimulation significantly elevated IL-8 and MIP-1β production compared to the LPS-negative control (*p* ≤ 0.05, data not included).

#### 3.1.3. Influence of Pre-Mycotoxin Exposure on LPS Immune Challenge

**Figure 4:**
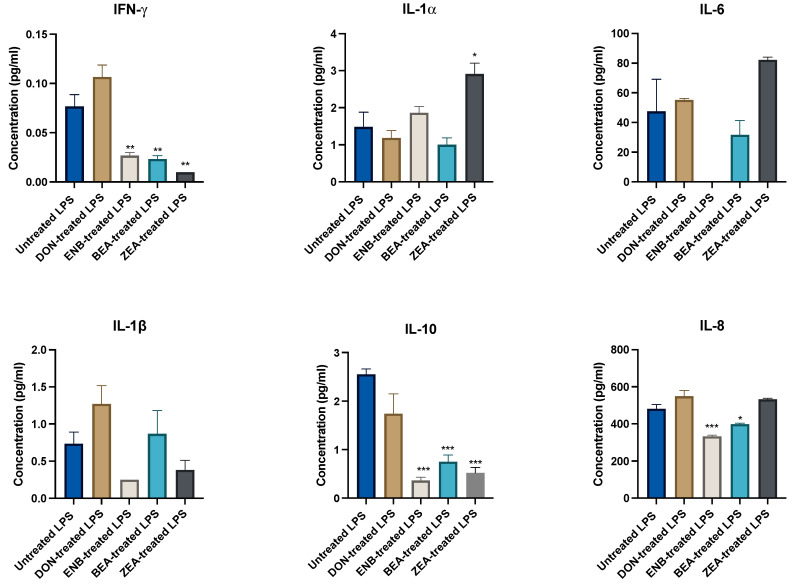
Effect of endotoxin (LPS for 24 h) challenge on cytokine and chemokine production post mycotoxins exposure for 48 h in the culture supernatant of the co-culture system (Bovine MAC-T cells and macrophages) compared to the untreated LPS control (no mycotoxin exposure) group. Data are expressed as mean (pg/mL) and SEM, and significant differences are denoted by * *p* < 0.05, ** *p* < 0.01 and *** *p* < 0.001. Includes results from deoxynivalenol (DON), enniatin B (ENB), beauvericin (BEA), and zearalenone (ZEN) exposure.

**Figure 5:**
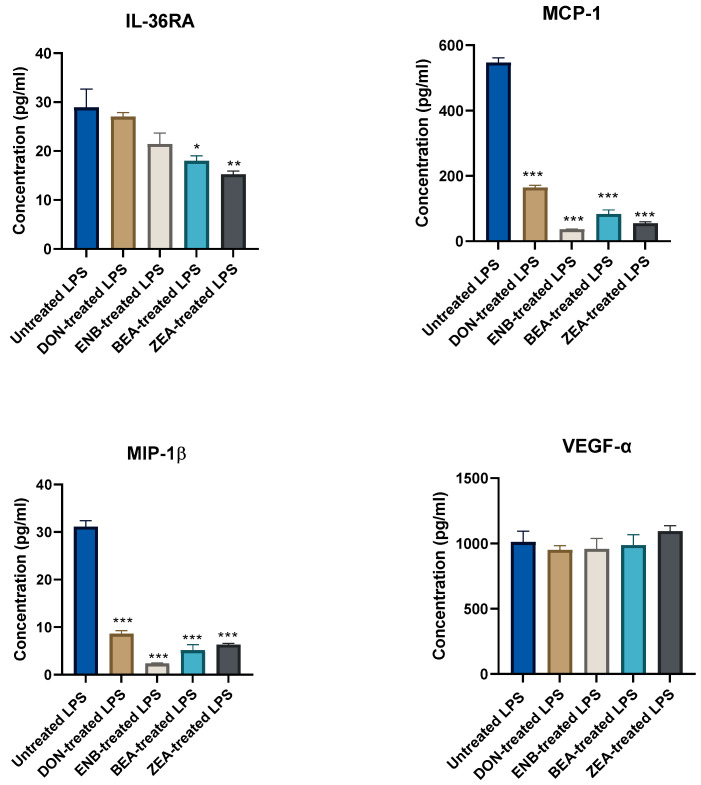
Effect of endotoxin (LPS for 24 h) challenge on cytokine and chemokine production post mycotoxins exposure for 48 h in the culture supernatant of the co-culture system (Bovine MAC-T cells and macrophages) compared to the untreated LPS control (no mycotoxin exposure) group. Data are expressed as mean (pg/mL) and SEM, and significant differences are denoted by * *p* < 0.05, ** *p* < 0.01 and *** *p* < 0.001. Includes results from deoxynivalenol (DON), enniatin B (ENB), beauvericin (BEA), and zearalenone (ZEN) exposure.

The influence of pre-mycotoxin exposure on LPS-induced inflammation was examined by comparing LPS-stimulated mycotoxin-treated groups with the LPS-positive control group without mycotoxin exposure, as illustrated in Figure 4 and Figure 5. The findings revealed a significant decrease in the production of IFN-γ, IL-8, IL-10, MCP-1, and MIP-1β following ENB exposure. Intriguingly, all mycotoxins significantly reduced MCP-1 and MIP-1β chemokine production (*p* ≤ 0.05). There were no significant changes observed in the concentrations of IL-1α, IL-6, IL-1β, and VEGF-α in the mycotoxin-treated groups. 

### 3.2. Identification of DEGs in MAC-T Cells Pre-Incubated with Mycotoxins Then Immune Challenged with LPS

Transcriptomic analysis using RNA-Seq was employed to examine the impact of DON, ENB, and BEA on LPS-induced inflammation in a co-culture system. The Mac-T cells were exposed to the mycotoxins for 48 h, followed by a 24 h immune challenge with LPS. Subsequently, total RNA was isolated and subjected to RNA-Seq analysis, enabling us to identify DEGs after the LPS challenge, with and without prior mycotoxin exposure.

**Figure 6:**
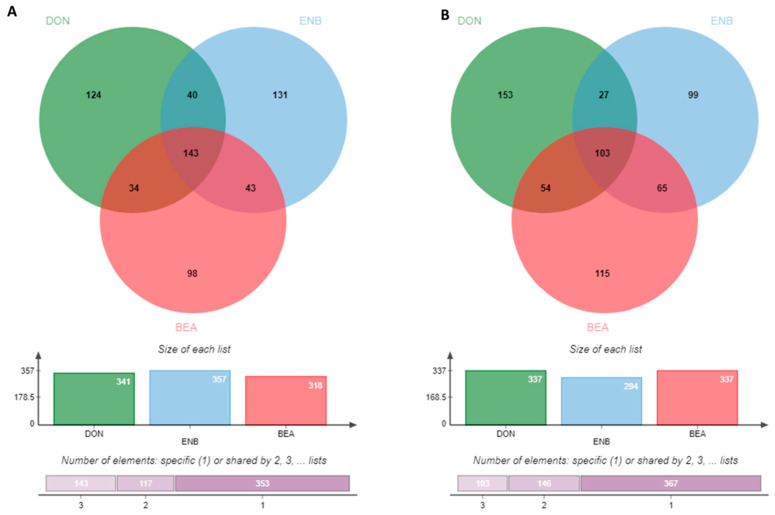
Venn diagram indicating differentially expressed up-regulated genes (**A**) and down-regulated genes (**B**) between Deoxynivalenol (DON), enniatin B (ENB) and beauvericin (BEA) treated groups as compared to the LPS control group in MAC-T cells of the co-culture system (Bovine MAC-T and macrophages) challenged with *E. coli* LPS for 24 h post mycotoxins exposure for 48 h.

The results of the transcriptomic analysis unveiled a noteworthy trend of up-regulated genes in the groups treated with mycotoxins plus LPS when compared to the LPS-positive control group. More specifically, the DON-treated group displayed 341 up-regulated genes, the ENB-treated group showed 357 up-regulated genes, and the BEA-treated group exhibited 318 up-regulated genes relative to the control group (as shown in Figure 6A). In contrast, the mycotoxin-treated groups demonstrated down-regulation in 337 genes for DON, 294 genes for ENB, and 337 genes for BEA (depicted in Figure 6B). To visually depict the overlap of differentially expressed genes (DEGs) across the various groups, a Venn diagram was constructed that revealed the presence of 143 common genes among the DON, ENB, and BEA exposure groups, constituting roughly 50% of the genes from each group (as illustrated in Figure 6). Additionally, 103 common DEGs were identified that displayed down-regulation in all three mycotoxin exposure groups.

### 3.3. Function Enrichment Analysis of Mac-T Cell DEGs

To further explore the immunomodulatory effects of DON, ENB and BEA, pathway enrichment analysis was carried out with the DEGs using the KEGG and GO databases. All significantly impacted pathways in Mac-T cells were identified using a threshold of *p* ≤ 0.05 in the KEGG enrichment analysis. The DEGs from Mac-T cells treated with DON, ENB and BEA exhibited enrichment in 8, 11, and 10 pathways, respectively (Table 1, Table 2 and Table 3). Notably, three signaling pathways were found to be commonly regulated by all three mycotoxins, indicating a partial overlap in their toxic effects on Mac-T cells. Additionally, among the pathways influenced by DON, ENB and BEA, three of the top pathways were consistent across all three mycotoxins, namely the apoptosis signaling pathway, inflammation mediated by chemokine and cytokine signaling pathway, and Wnt signaling pathway. Interestingly, the Toll receptor signaling pathway was specifically induced by the ENB and BEA treatment groups.

## 4. Discussion

Mycotoxins have been shown to affect immune-related organs and cells, influencing host defenses against infectious agents and related microbial toxins [27]. In vitro cellular systems, such as the mammary epithelial MAC-T cell line [28] and other cell types, have been widely utilized as effective methods for assessing mycotoxicity [29,30]. Additionally, macrophages have been employed as an in vitro cell model to evaluate the potential impact of mycotoxins on the immune system [31,32], given their crucial roles as phagocytic antigens presenting in and repairing cells of the innate immune system [33]. 

Preserving the integrity of barrier functions and ensuring the effective operation of both innate and adaptive immune responses are vital for combatting infectious pathogens and harmful metabolites. The potential disruption of these mechanisms may arise from fungal cell wall antigens and the immunomodulatory or immunotoxic effects of mycotoxins, potentially contributing to the onset or aggravation of immune-related disorders. Additionally, Gram-negative bacteria have the capacity to trigger diverse pathological effects through their cell wall-derived LPS endotoxins. Therefore, our study was designed to explore how mycotoxin exposure affects LPS-induced inflammation. We employed an in vitro co-culture model, consisting of bovine MAC-T cells and macrophages, for this investigation. We evaluated cytokine and chemokine concentrations in the co-culture supernatants following mycotoxin exposure, followed by an immune challenge using *E. coli* LPS, employing multiplex immunoassays. Additionally, we conducted comprehensive transcriptome analyses of the MAC-T cells within the co-culture system that had been exposed to mycotoxins.

The presence of mycotoxins in the food chain poses a potential hazard to the health of both humans and animals due to their toxic properties and high stability under heat treatment. In our current study, we focused on investigating the immunological effects of *Fusarium* mycotoxins, specifically deoxynivalenol (DON), zearalenone (ZEA), enniatin B (ENB), and beauvericin (BEA). We found significant changes in cytokine production within the mycotoxin treatment groups exposed to LPS compared to their respective controls without LPS treatment. Following exposure to DON, for example, LPS significantly stimulated the production of IFN-γ, IL-1α, IL-8, MCP-1, MIP-1β, and VEGF-α. These findings suggest that the damage caused by DON exposure to the barrier and tight junction of the epithelial monolayer may facilitate the diffusion of LPS through the epithelial layer to macrophages in the lower compartment of the co-culture. In support of this hypothesis, our previous research reported that DON, ENB, and BEA differentially disrupted the paracellular permeability of MAC-T cells [34]. The maintenance of intact barrier functions, as well as innate and adaptive immune responses, is crucial for effectively eliminating infectious pathogens and toxic metabolites. It has been previously demonstrated that ENB and BEA can also decrease the trans-epithelial electrical resistance (TEER) reading in IPEC-J2 cells after 48 h of exposure [35].

Innate and adaptive immunity play vital roles in controlling and eliminating microbial infections. Mucosal barriers, in conjunction with sentinel and effector cells, serve to protect underlying tissues from pathogens and actively participate in microbial recognition and defense. The production of cytokines and alarmins at mucosal surfaces drives the recruitment of specialized innate and adaptive immune cells, such as macrophages, neutrophils, dendritic cells, and T cells, to combat invading microbes. Unfortunately, most mycotoxins have a detrimental effect on the structural integrity of mucosal barriers and can suppress cellular immunity depending on the dose of the mycotoxin exposure [36]. Consequently, mycotoxin-induced alterations in innate and adaptive host defenses can increase susceptibility to infection [37]. The comprehensive study by Bahrami et al. in 2016 extensively documented the notable influence of mycotoxins on the integrity of epithelial barriers [38]. 

Whole-transcriptome analysis holds a pivotal role in unraveling the genetic architecture and functionality of genomes, revealing cellular genetic networks, and shedding light on aspects of physiology, biochemistry, and biological systems. It also aids in the identification of molecular biomarkers responsive to both pathogens and environmental challenges. In this particular study, we employed RNA-Seq to scrutinize genome-wide expression profiles of MAC-T cells after an immune challenge with LPS, both before and after exposure to mycotoxins. Our comparative analyses involved groups such as LPS vs. Control and mycotoxin-LPS vs. LPS Control. These comparisons were instrumental in elucidating distinct biological mechanisms between mycotoxin-induced and LPS-induced differential gene expression, offering valuable insights into how prior mycotoxin exposure might impact the development of endotoxemia induced by *E. coli* LPS. Additionally, we conducted enrichment analyses, including GO and KEGG analyses, to systematically identify genes associated with cellular responses to stimuli and signaling in LPS-challenged MAC-T cells following mycotoxin exposure, as these processes were naturally overrepresented. Employing whole-RNA transcriptome sequencing, we explored differentially expressed genes (DEGs) potentially involved in the inflammatory signaling pathway and oxidative stress-related pathway in bovine mammary epithelial cells stimulated by LPS [39,40]. In the present study, both GO-enriched terms and KEGG pathways associated with inflammation mediated by chemokines and cytokine signaling pathway response, apoptosis signaling pathway, and Wnt signaling pathway were the most relevant DEGs enriched in LPS-treated MAC-T cell post all three mycotoxin exposures, indicating that mycotoxins had appreciable impacts on inflammation and apoptosis. 

Mycotoxins, being xenobiotics, undergo a multi-phasic metabolic detoxification process orchestrated by a coordinated series of enzymatic actions [41]. In the initial “modification” phase, these xenobiotics are transformed into intermediary metabolites. Subsequently, these intermediates are “conjugated” by a wide array of transferases, preparing them for “excretion” via efflux membrane transporters. While the general detoxification process is reasonably well understood, the signaling pathways responsible for determining specific responses to various classes of mycotoxins remain less defined. In the context of our current study, the differentially expressed genes (DEGs) and GO terms indicated that exposure to all three mycotoxins significantly influenced the Wnt signaling pathway. This observation aligns with previous findings suggesting that canonical Wnt signaling may play a role in detoxification triggered by exposure to mycotoxins of the epipolythiodioxopiperazine (ETP) class, with Akt activation being noted as well [42]. 

The RNA-Seq results provided a comprehensive overview of the gene expression patterns within MAC-T cells under the influence of DON, ENB, and BEA. Noticeable alterations were observed when comparing the mycotoxin-exposed groups to the control groups. Interestingly, the number of up-regulated and down-regulated genes was nearly equivalent. Furthermore, the gene expression profiles highlighted the variability of differentially expressed genes (DEGs) between the ENB and BEA-treated groups, with even commonly shared genes exhibiting distinct expression patterns in these two sets. Specifically, the ENB-treated group displayed a higher number of up-regulated DEGs compared to the BEA-treated group, totaling 357 and 318, respectively, implying that ENB exerts a more potent stimulus during exposure. As depicted in the Venn diagrams, a substantial portion of DEGs in each group was found to be regulated by all three mycotoxins, suggesting that the toxic effects of these three mycotoxins are parallel in MAC-T cells. Moreover, the enrichment analysis of the RNA-Seq data revealed that DON, ENB, and BEA collectively influence a wide array of pathways, including those related to chemokine-mediated inflammation, cytokine signaling responses, apoptosis signaling, coenzyme A biosynthesis, and Wnt signaling.

## 5. Conclusions

In conclusion, this study established a co-culture system of bovine mammary epithelial cells (MAC-T) and macrophages (BoMac) to evaluate the impact of mycotoxin exposure on LPS-induced inflammation. The results from multiplex cytokine analysis indicated that DON exhibited a more pronounced effect on the inflammatory response when compared to ENB and BEA. However, RNA-Seq analysis revealed that all three mycotoxins shared similar effects on MAC-T cells, notably inducing inflammatory, apoptotic signaling, and the Wnt signaling pathways. Collectively, this study provides valuable preliminary empirical insights into the molecular mechanisms underlying inflammation triggered by mycotoxin exposure, with a particular emphasis on *Fusarium* class mycotoxins. These findings contribute to a deeper comprehension of how early exposure to mycotoxins can potentiate the inflammatory response to pathogens, particularly in the presence of LPS. 

## Figures and Tables

**Table 1:** Deoxynivalenol (DON)-induced Kyoto Encyclopedia of Gene and Genome (KEGG) pathways of the DEGs in MAC-T cells co-cultured with macrophages.

Panther Pathways	Bos Taurus-REFLIST	Upload (402)	Upload (Expected)	Upload (over/under)	Upload (Fold Enrichment)	Upload (Raw *p*-Value)
Methylmalonyl pathway	9	3	0.16	+	18.9	1.01 × 10^−3^
Coenzyme A biosynthesis	9	2	0.16	+	12.6	1.49 × 10^−2^
Axon guidance mediated by netrin	37	5	0.65	+	7.66	7.65 × 10^−4^
Opioid prodynorphin pathway	39	3	0.69	+	4.36	3.60 × 10^−2^
Apoptosis signaling pathway	135	8	2.38	+	3.36	3.54 × 10^−3^
T cell activation	92	5	1.62	+	3.08	2.69 × 10^−2^
Inflammation mediated by chemokine and cytokine signaling pathway	261	10	4.6	+	2.17	2.89 × 10^−2^
Wnt signaling pathway	294	11	5.18	+	2.12	2.26 × 10^−2^
Unclassified (UNCLASSIFIED)	19,997	333	352.61	-	0.94	4.59 × 10^−3^

**Table 2:** Enniatin B (ENB)-induced Kyoto Encyclopedia of Gene and Genome (KEGG) pathways of the DEGs in MAC-T cells co-cultured with macrophages.

Panther Pathways	Bos Taurus-Reflist (22,798)	Upload (416)	Upload (Expected)	Upload (over/under)	Upload (Fold Enrichment)	Upload (Raw *p*-Value)
Plasminogen activating cascade	22	7	0.4	+	17.44	6.27 × 10^−7^
Adenine and hypoxanthine salvage pathway	12	2	0.22	+	9.13	2.53 × 10^−2^
Axon guidance mediated by netrin	37	4	0.68	+	5.92	6.10 × 10^−3^
Alzheimer disease-presenilin pathway	126	12	2.3	+	5.22	7.47 × 10^−6^
Toll receptor signaling pathway	65	5	1.19	+	4.22	8.42 × 10^−3^
Apoptosis signaling pathway	135	9	2.46	+	3.65	1.19 × 10^−3^
T cell activation	92	5	1.68	+	2.98	3.04 × 10^−2^
Integrin signaling pathway	194	9	3.54	+	2.54	1.13 × 10^−2^
Inflammation mediated by chemokine and cytokine signaling pathway	261	12	4.76	+	2.52	3.99 × 10^−3^
CCKR signaling map	182	8	3.32	+	2.41	2.16 × 10^−2^
Wnt signaling pathway	294	12	5.36	+	2.24	1.34 × 10^−2^
Unclassified (UNCLASSIFIED)	19,997	333	364.89	-	0.91	1.16 × 10^−5^

**Table 3:** Beauvericin (BEA)-induced Kyoto Encyclopedia of Gene and Genome (KEGG) pathways of the DEGs in MAC-T cells co-cultured with macrophages.

Panther Pathways	Bos Taurus-REFLIST (22,798)	Upload (380)	Upload (Expected)	Upload (over/under)	Upload (Fold Enrichment)	Upload (Raw *p*-Value)
Toll pathway-drosophila (P06217)	2	1	0.03	+	30	4.84 × 10^−2^
Coenzyme A biosynthesis (P02736)	9	2	0.15	+	13.33	1.34 × 10^−2^
Plasminogen activating cascade (P00050)	22	4	0.37	+	10.91	7.99 × 10^−4^
Adenine and hypoxanthine salvage pathway (P02723)	12	2	0.2	+	10	2.14 × 10^−2^
Axon guidance mediated by netrin (P00009)	37	4	0.62	+	6.49	4.46 × 10^−3^
Toll receptor signaling pathway (P00054)	65	5	1.08	+	4.61	5.84 × 10^−3^
Apoptosis signaling pathway (P00006)	135	9	2.25	+	4	6.34 × 10^−4^
T cell activation (P00053)	92	6	1.53	+	3.91	5.55 × 10^−3^
Alzheimer disease-presenilin pathway (P00004)	126	7	2.1	+	3.33	6.43 × 10^−3^
Wnt signaling pathway (P00057)	294	13	4.9	+	2.65	1.79 × 10^−3^
Inflammation mediated by chemokine and cytokine signaling pathway (P00031)	261	10	4.35	+	2.3	1.44 × 10^−2^
Unclassified (UNCLASSIFIED)	19,997	306	333.31	-	0.92	7.78 × 10^−5^

## Data Availability

The datasets used and analyzed during the current study are available from the corresponding author upon request.
